# Supplementary material for: The Association of Meteorological Factors with Cognitive Function in Older Adults
Source: Int J Environ Res Public Health. 2021 Jun 2;18(11):5981. doi: 10.3390/ijerph18115981 (PMC8199712; doi:10.3390/ijerph18115981)
Supplement: Supplementary file 1 [file ijerph-18-05981-s001.zip › ijerph-1231051-supplementary.pdf]

Supplementary materials

As shown in *Figure S1* and *Table S1*, we checked the statistical assumptions, the results showed that the samples in this study conformed to the normal distribution, and there was not strong multicollinearity among those variables.

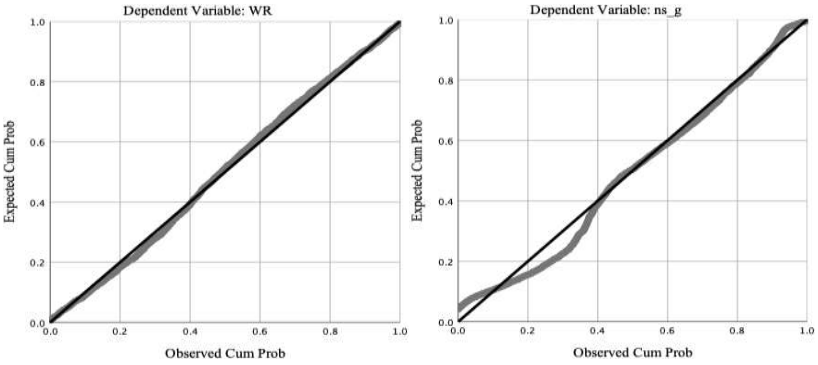

Figure S1. Normal P-P plot of regression standardized residual.

Table S1. Analysis of multicollinearity by stepwise regression.

Variable	Collinearity statistics					
	The memory test			The logical sequence test		
	Beta	Tolerance	VIF	Beta	Tolerance	VIF
Age	-.222	.994	1.006	-.155	.990	1.010
Gender	.058	.975	1.025	-.121	.973	1.028
Residence	-.106	.973	1.028	-.124	.964	1.038
Chronic disease	-.017	.988	1.012	.031	.987	1.013
Temperature	.023	.872	1.146	.091	.989	1.012
Humidity	-.065	.997	1.003	-.015	.878	1.139

In logic sequence test, we consider the ICC and design effect values of each model. The fitting degree of these models was too low to use the mixed model for further analysis. We used a simpler single-layer linear regression analysis method, which was more convenient and the results were reliable. Therefore, we put the full model results in the supplementary materials. As shown in *Table S2*.

Table S2. The effects of individual level and provincial level variable in the full model.

Parameter	Estimate	*SE*	*df*	*t*	*p*
Intercept	19.783052	11.308369	3431.962	1.749	.080
Individual level variable					
Age	-.163244	.152287	3419.972	-1.072	.284
Gender	-.750802	1.865086	3417.361	-.403	.687
Residence	-1.132281	1.839438	3240.528	-.616	.538
Chronic disease	-1.919087	1.824181	3418.348	-1.052	.293
Provincial level variable					
Temperature	-.518626	.217403	3417.695	-2.386	.017
Humidity	.093173	.156729	3431.559	.594	.552
Control variable					
Traffic	4.184973E-6	7.836733E-6	17.315	.534	.600
Provincial level moderating effects					

Age × Temperature	.005352	.002900	3423.288	1.845	.065
Age × Humidity	-.000964	.002107	3420.032	-.457	.648
Gender × Temperature	.043313	.035918	3424.586	1.206	.228
Gender × Humidity	-.018285	.025636	3421.073	-.713	.476
Residence × Temperature	.096909	.038770	3250.920	2.500	.012
Residence × Humidity	-.027500	.026039	3168.618	-1.056	.291
Chronic disease × Temperature	.001713	.037996	3421.857	.045	.964
Chronic disease × Humidity	.028350	.025356	3417.003	1.118	.264
